# The effect of time pressure and ego depletion on young children’s helping behavior

**DOI:** 10.3389/fpsyg.2025.1584248

**Published:** 2025-04-17

**Authors:** Zihan Zha, Chang Chen, Renyuan Zhang, Wenjie Zhang

**Affiliations:** ^1^Faculty of Education and Science, Hunan Normal University, Changsha, China; ^2^Cognition and Human Behavior Key Laboratory, Hunan Normal University, Changsha, China; ^3^Institute of Psychology, Chinese Academy of Sciences, Beijing, China

**Keywords:** helping behavior, time pressure, ego depletion, dual-process theory, cognitive resources

## Abstract

Based on the theory of limited cognitive resources and the dual-process theory, this study explores the mechanisms by which time pressure (an external factor) and ego depletion (an internal factor) affect helping behavior in children aged 4 to 6 years through two experiments. Experiment 1 (*n* = 153, *M* = 5.42 years, *SD* = 0.71) examined the impact of time pressure on children’s helping behavior, while Experiment 2 (*n* = 221, *M* = 5.41 years, *SD* = 0.76) investigated the role of ego depletion. The results revealed that both time pressure and ego depletion significantly inhibited children’s helping behavior, with notable age-related differences in these effects: the helping behavior of 4- and 5-year-old children was significantly reduced under conditions of time pressure and resource depletion, whereas 6-year-old children demonstrated greater resistance to these disruptions. These findings suggest that children’s helping behavior relies more on the cognitive processing of the deliberative system rather than the automatic responses of the intuitive system. Furthermore, as children grow older, they gradually develop more effective cognitive resource regulation abilities to counteract the negative effects of resource depletion.

## Introduction

### Children’s helping behavior

Prosocial behavior are social actions that provide other people with resources, instrumental help, comfort, or the expression of empathic/sympathetic feelings (Hay et al., 2021), including helping, sharing, and comforting (Dunfield, 2014). As a typical manifestation of prosocial behavior, helping behavior refers to actions in which an individual actively assists others in resolving difficulties without prior commitment to receiving rewards (Eisenberg et al., 2013). Research has shown that helping behavior not only enhances young children’s awareness of serving others and society but also improves interpersonal relationships and adaptability (Brownell et al., 2013; Aknin et al., 2012). Studies indicate that even during infancy, children exhibit concern for others’ difficulties and a tendency to offer help (Warneken and Tomasello, 2006). By around the age of 2, young children can understand others’ goals and provide instrumental assistance. By age 3, children begin to exhibit context-specific helping responses (Paulus, 2014). Four-year-olds are able to understand others’ needs and respond with appropriate help (Martin and Olson, 2013), while 5-year-olds demonstrate goal-directed, proactive helping and consider others’ specific needs (Sierksma and Shutts, 2021). By age 6, children’s helping behavior becomes more stable, selective, and targeted (Paulus et al., 2020). These findings suggest that the ages of 4 to 6 represent a critical period for the formation of helping behavior patterns in young children, with notable age-related changes in behavioral characteristics.

Prosocial behavior, including helping behavior, is influenced by various factors that can be broadly categorized into internal and external factors. Internal factors primarily refer to individual characteristics, such as age, gender, cognitive abilities, emotional states, and personality traits (Eisenberg et al., 2006). Self-control ability is another critical internal factor affecting prosocial behavior. Studies have shown that ego depletion reduces individuals’ tendency to engage in prosocial behavior (Meng and Moriguchi, 2021). From the perspective of the theory of limited cognitive resources, cognitive resources are finite, and cognitive processing requires the consumption of these resources (Baumeister and Vohs, 2018). When cognitive resources are occupied or depleted, individuals’ cognitive and executive functions are significantly impaired (Diamond, 2013). External factors, such as time pressure, group relationships, and social support, also influence prosocial decision-making (Plötner et al., 2021; Katerkamp and Horn, 2025). The dual-process theory divides human decision-making into two systems: the fast, automatic, and heuristic-driven intuitive system, and the slow, deliberate, and reflective deliberative system (Evans and Stanovich, 2013). The aforementioned internal and external factors that influence prosocial behavior operate through different mechanisms on the intuitive and deliberative systems.

Ages 4 to 6 represent a critical period for the development of cognitive abilities and self-control in young children (Pushparatnam et al., 2021), as well as a transitional stage where helping behavior shifts from being context-dependent to actively regulated. A key debate in current research centers on whether young children’s helping behavior is driven by intuitive responses or requires the mobilization of cognitive resources to suppress selfish impulses. This study focuses on children aged 4 to 6 and, by integrating the theory of limited cognitive resources and the dual-process theory, systematically explores the mechanisms influencing helping behavior from two dimensions: time pressure (external) and ego depletion (internal). The aim is to uncover the roles of the intuitive system and the deliberative system in young children’s helping behavior decisions.

### Time pressure and prosocial behavior in young children

Time pressure refers to the anxiety experienced by decision-makers as they perceive an increasing urgency to complete a task (Sinha and Smith, 2000). Research has shown that time pressure can influence prosocial behavior by either activating the intuitive system or inhibiting the deliberative system (Evans and Curtis-Holmes, 2005). It may affect prosocial behavior through two mechanisms: first, by activating the intuitive system, which accelerates instinctive prosocial responses (Corbit et al., 2023; Plötner et al., 2021); and second, by inhibiting the deliberative system, as cognitive resource depletion hinders altruistic decision-making (Steinbeis, 2018).

In studies on adults, the effects of time pressure on prosocial behavior have yielded contradictory findings. Some research suggests that time pressure promotes prosocial behavior. For example, Rand et al. (2012) found that participants under time pressure exhibited greater tendencies toward cooperation in economic game tasks. Similarly, Everett et al. (2017) observed that time pressure increased participants’ willingness to cooperate with others. However, other studies have reported opposite results: Gärtner (2018) found that time pressure led to more selfish choices, while Jarke-Neuert and Lohse (2022) demonstrated that time pressure increased selfish behavior by reinforcing information avoidance. In research on children, Plötner et al. (2021) found that children aged 3–7 shared more stickers under time pressure than in delayed conditions. Similarly, Corbit et al. (2023) reported that children aged 7–12 were more likely to choose cooperation under time pressure. Notably, Nava et al. (2023) explored the impact of time pressure on the cooperative behavior of adolescents and adults, finding that adolescents exhibited a pattern opposite to that of adults, with time pressure reducing their cooperative behavior.

Although previous studies have explored the effects of time pressure on prosocial behavior, its underlying mechanisms remain insufficiently validated in the domain of helping behavior. Existing research has primarily focused on sharing and cooperative behaviors (Plötner et al., 2021), whereas helping behavior, due to its higher spontaneity and contextual dependence, may be influenced by time pressure through fundamentally different pathways. Grounded in the dual-process theory of decision-making (Evans and Stanovich, 2013; Lohse et al., 2017), this study systematically examines the mechanisms by which time pressure affects helping behavior in children aged 4–6, addressing the following core questions: Does time pressure reduce helping behavior in young children by inhibiting the deliberative system, or does it promote helping decisions by engaging the intuitive system? Drawing on the theory of limited cognitive resources (Baumeister and Vohs, 2018), we propose the following hypothesis: Time pressure inhibits the deliberative system, resulting in cognitive resource depletion, which significantly reduces helping behavior in young children (H1).

### Self-control resources and prosocial behavior in young children

Cognitive resources refer to the limited mental resources required by individuals to complete tasks, including attention, memory, and executive function (Baumeister, 2002; Shah et al., 2012). Self-control resources, as a critical component, are specifically used to inhibit impulses and regulate behavior to conform to social norms, often metaphorically referred to as the “moral muscle” (Baumeister and Vohs, 2018). Researchers, using methods such as the Stop-Signal Task (Meng and Moriguchi, 2021) and the dual-task paradigm (Ugur, 2021), have identified the limited and depletable nature of self-control resources.

In research on adults, the conclusion that ego depletion significantly reduces prosocial behavior has been widely supported. For example, in dictator games, resource depletion reduces the amount allocated to others by 20–30% (Halali et al., 2013), while in helping scenarios, response times increase by 1.5 times (DeWall et al., 2008). Moreover, the depletion of self-control resources may also lead individuals to exhibit more self-serving behavior when faced with complex social situations (Ugur, 2021).

In research on children, the findings remain inconsistent. Children aged 4–6, whose cognitive resources—particularly self-control abilities—are not yet fully developed, are more susceptible to the effects of cognitive resource depletion on their prosocial behavior (Carlson et al., 2013). Studies have shown that after completing inhibitory control tasks, the frequency of children’s sharing behavior drops to 42% of baseline levels (Meng and Moriguchi, 2021). This depletion may operate through dual pathways: at the cognitive level, it weakens emotional recognition and theory of mind abilities (Wellman and Liu, 2004); at the behavioral level, it reduces empathic responses and triggers indifference or avoidance (Eisenberg et al., 1998). Notably, most existing research focuses on sharing and cooperative behavior, while helping behavior, due to its goal-directed nature (requiring active recognition of others’ needs) and executional complexity (requiring the coordination of multi-step actions), may be even more sensitive to resource depletion (Paulus, 2014).

Although existing research has made significant progress, most studies have focused on sharing and cooperative behaviors, with relatively little attention given to helping behavior. This makes it difficult to clarify whether resource depletion influences helping decisions through the intuitive system or the deliberative system. Grounded in the dual-process theory (Evans and Stanovich, 2013), this study systematically examines the mechanisms by which ego depletion affects helping behavior in children aged 4–6. It seeks to address the following core questions: Does ego depletion reduce helping behavior in young children by inhibiting the deliberative system? Or, alternatively, do children rely on the intuitive system to promote helping decisions under conditions of resource depletion? On this basis, combined with the theory of limited cognitive resources (Baumeister and Vohs, 2018), this study proposes the following hypothesis: Ego depletion significantly reduces the frequency of children’s helping behavior by inhibiting the deliberative system (H2).

### Current study

Existing research on the mechanisms influencing helping behavior in young children reveals several limitations. First, the effect of time pressure on prosocial behavior shows contradictory findings, and there is a lack of direct investigation into helping behavior among 4–6-year-old children (Rand et al., 2012; Plötner et al., 2021). Second, the role of self-control resources in helping behavior remains unclear, as most studies have focused on sharing behaviors while neglecting a systematic exploration of helping behaviors (Meng and Moriguchi, 2021; Ugur, 2021). Finally, previous studies have yet to reach a consensus on whether helping behavior in young children is driven by intuitive responses or requires the mobilization of cognitive resources for deliberative decision-making (Rand et al., 2012).

This study focuses on children aged 4–6 to explore the mechanisms by which time pressure (an external factor) and ego depletion (an internal factor) influence helping behavior. It aims to address the following core questions: (1) How does time pressure affect young children’s helping behavior? (2) How does ego depletion impact young children’s helping behavior? Based on the theory of limited cognitive resources (Baumeister and Vohs, 2018), the following hypotheses are proposed:

*H1:* Time pressure inhibits the deliberative system, leading to cognitive resource depletion, which in turn reduces helping behavior in young children.

*H2:* Ego depletion inhibits the deliberative system, thereby decreasing helping behavior in young children.

*H3:* As children grow older, their helping behavior gradually increases.

Therefore, this study focuses on children aged 4–6, integrating the theory of limited cognitive resources and the dual-process theory of decision-making. It aims to explore the mechanisms by which time pressure (an external factor) and ego depletion (an internal factor) influence helping behavior in young children. The study seeks to address the theoretical question of whether young children’s helping behavior relies more on the intuitive system or the deliberative system.

## Experiment 1: the impact of time pressure on helping behavior in 4–6-year-old children

### Purpose

Experiment 1 combines a time pressure task with the classic pen-picking helping paradigm to examine the differences in helping behavior among 4-, 5-, and 6-year-old children under conditions of external time pressure and no time pressure. The aim is to reveal the mechanism by which time pressure affects helping behavior in children aged 4–6.

### Methods

#### Participants

Using G*Power 3.1 (Faul et al., 2009), it was calculated that 128 participants would be required to ensure sufficient statistical power (1–β > 0.8) under a medium effect size (*f* = 0.25). This study recruited a total of 153 participants aged 4 to 6 years from a region in central China. Specifically, our sample included 54 4-year-olds (*M* = 4.62 years, *SD* = 0.25 years), 51 5-year-olds (*M* = 5.47 years, *SD* = 0.29 years), and 48 6-year-olds (*M* = 6.25 years, *SD* = 0.15 years). All participants were randomly assigned to either the time pressure group or the time delay group. Informed consent was obtained from the parents or guardians of all participants, and the study was approved by the ethics committee of the institution.

#### Experimental design

Experiment 1 used a 2 (Time Pressure: time pressure condition, time delay condition) × 3 (Age: 4, 5, 6) between-subjects experimental design. Time pressure and age served as the independent variables, while the dependent variable was the score of children’s helping behavior.

#### Procedure

**Figure 1:**
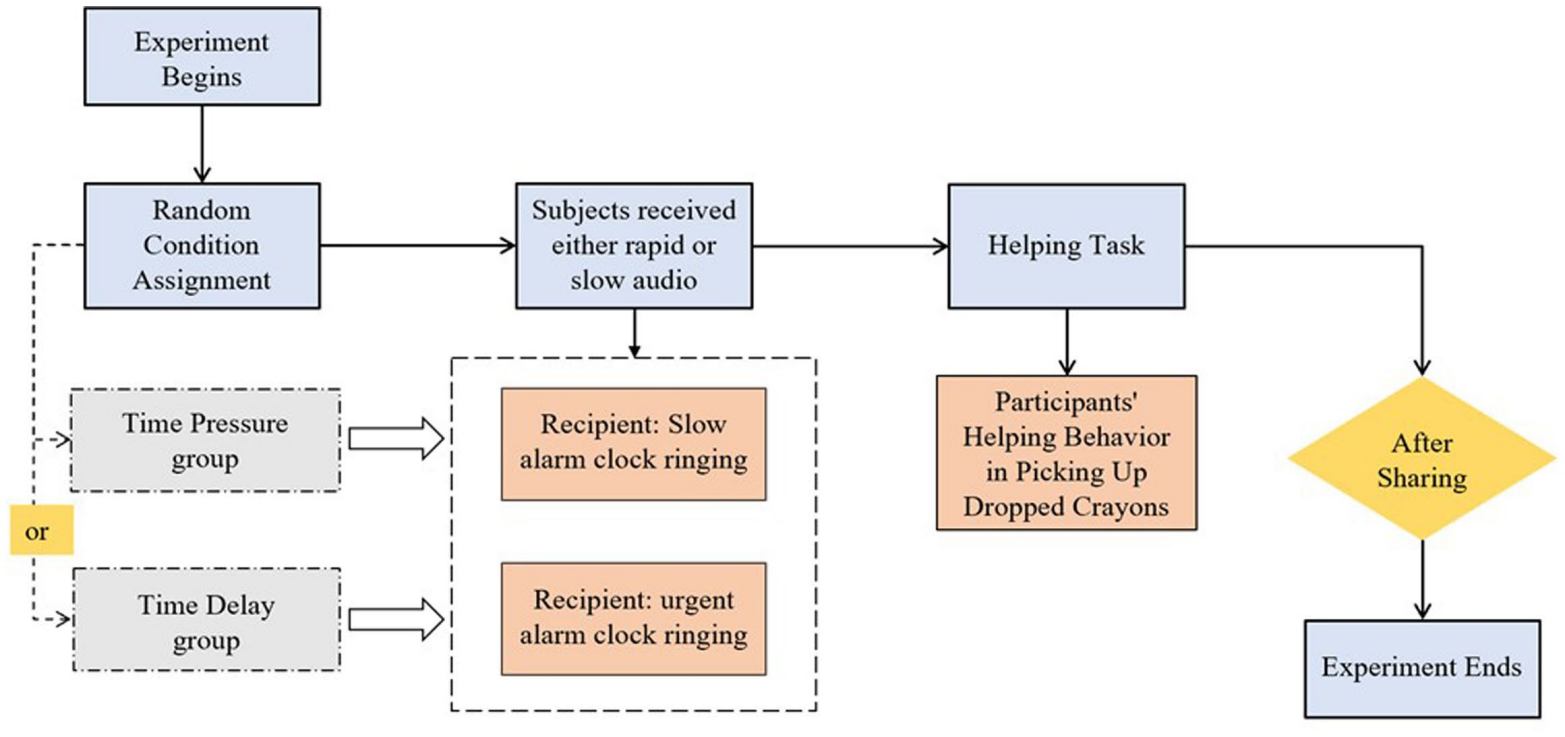
Flowchart of experiment 1.

This study adopted a time pressure paradigm adapted from Plötner et al. (2021), which consisted of the following steps (Figure 1):

1. Preparation: The testing environment was set up in a quiet laboratory, equipped with a timer, a recording device, and a transparent plastic box containing 12 colored crayons. Before the experiment began, the experimenter informed the children and their teachers that the procedure would take approximately 15 min.
2. Grouping: The children were randomly assigned to either the time pressure group or the time delay group.
3. Time Pressure Manipulation: The time pressure group was exposed to a fast-paced alarm sound accompanied by a countdown audio: “Your ride is leaving in 10 s, you need to hurry, 10, 9, 8... 3, 2, 1” (repeated twice). The time delay group, on the other hand, was exposed to a calm alarm sound accompanied by a counting-up audio: “Your ride will not arrive for a long time, you have plenty of time, 1, 2, 3... 8, 9, 10” (repeated twice).
4. Helping task: The experimenter said, “Alright, today’s task is done. I’ll tidy up, and then we’ll head back to the classroom!” The experimenter then “accidentally” knocked over the container of crayons while tidying the desk and expressed surprise: “Oh no!” The experimenter continued tidying the desk for 20 s without directly asking for help. If the child did not spontaneously help, the experimenter began slowly picking up the crayons for 10 s while muttering to themselves: “Oh, all the crayons fell on the floor. How clumsy of me.”
5. Conclusion: The experimenter thanked each child and gave them a sticker as a token of appreciation, ensuring they were in a positive mood before returning them to their classroom. All experimental procedures were conducted by two trained experimenters to ensure standardization and consistency throughout the study.

#### Data processing and analysis

Data Coding: providing help spontaneously while the experimenter was tidying the desk was scored as 1 point; providing help after the experimenter began picking up the crayons was scored as 0.5 points; failing to provide any help was scored as 0 points.

Data Analysis: All data from experiment 1 were processed and analyzed using R software (version 4.4.2). A two-way analysis of variance (Two-Way ANOVA) was conducted to examine the main effects and interaction effects of age (4 years, 5 years, 6 years) and time pressure (time pressure condition, time delay condition) on children’s helping behavior scores. If the ANOVA revealed significant main effects or interaction effects (*p* < 0.05), Tukey’s HSD was used for *post hoc* multiple comparisons. Additionally, to further explore the predictive effects of age (as a continuous variable) and time pressure on children’s helping behavior, multiple linear regression analysis was performed.

### Results

#### Preliminary data analysis

In the time pressure group, helping behavior scores increased with age, with 6-year-olds scoring higher (*M* = 0.58, *SD* = 0.43) than 5-year-olds (*M* = 0.44, *SD* = 0.45) and 4-year-olds (*M* = 0.34, *SD* = 0.43). A similar age-related trend was observed in the time delay group, where 6-year-olds achieved the highest scores (*M* = 0.81, *SD* = 0.33), followed by 5-year-olds (*M* = 0.70, *SD* = 0.43) and 4-year-olds (*M* = 0.60, *SD* = 0.47), as shown in Table 1.

**Table 1:** Descriptive statistics of the outcome of young children’s helping behavior under different time pressure conditions.

Age group	Time pressure type	*n*	*M* ± *SD*
4 years old group	Time pressure group	28	0.339 ± 0.431
	Time delay group	26	0.596 ± 0.469
5 years old group	Time pressure group	26	0.442 ± 0.454
	Time delay group	25	0.700 ± 0.433
6 years old group	Time pressure group	24	0.583 ± 0.434
	Time delay group	24	0.812 ± 0.323

#### Analysis of variance

**Figure 2:**
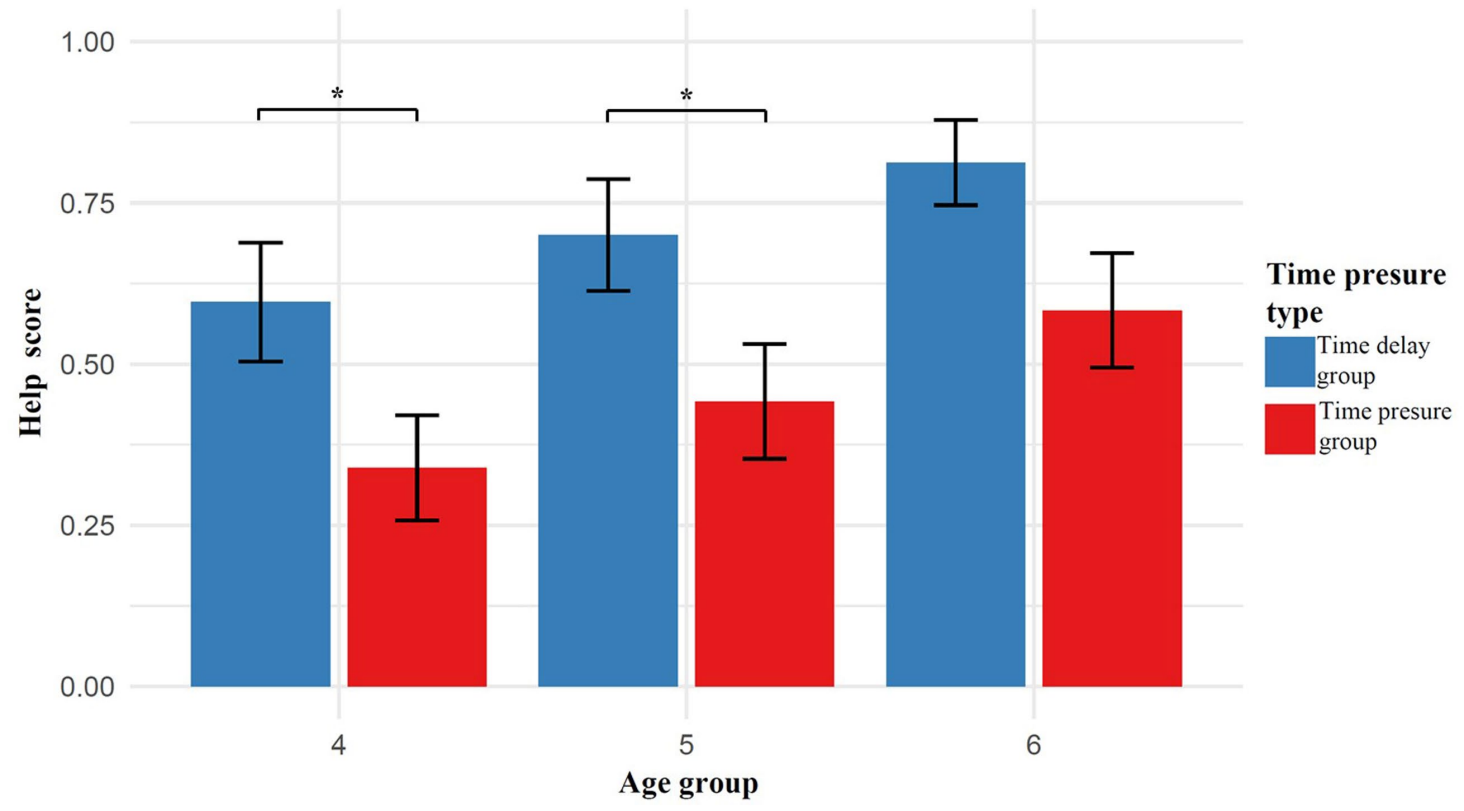
Differences in helping behavior of children under different time pressure conditions.

Using helping behavior scores as the dependent variable, a two-way analysis of variance (ANOVA) with 2 time pressure conditions (time pressure group, time delay group) × 3 age groups (4, 5, 6) was conducted. The results showed that age had a significant effect on helping scores, with a significant main effect of age, *F*(2, 150) = 3.83, *p* = 0.024, η^2^ₚ = 0.050. *Post hoc* multiple comparisons revealed that the helping behavior of 4-year-olds was significantly lower than that of 6-year-olds (*p* = 0.023), while no significant differences were found between 5-year-olds and 4-year-olds (*p* = 0.654) or between 5-year-olds and 6-year-olds (*p* = 0.429). The main effect of time pressure was also significant, *F*(1, 151) = 12.88, *p* < 0.001, η^2^ₚ = 0.081, indicating a highly significant difference between the time pressure group and the time delay group (*p* < 0.001). However, the interaction effect between time pressure and age was not significant, *F*(2, 147) = 0.018, *p* = 0.982, η^2^ₚ = 0.002 (Figure 2).

Simple effects analysis revealed no significant differences in helping behavior among 4-year-olds, 5-year-olds, and 6-year-olds under both the time pressure and time delay conditions (*ps* > 0.05). However, further simple effects analysis showed that, within the 4-year-old group, children in the time delay condition (*M* = 0.60, *SD* = 0.47) exhibited significantly higher helping behavior than those in the time pressure condition (*M* = 0.34, *SD* = 0.43), *p* = 0.029. Similarly, within the 5-year-old group, children in the time delay condition (*M* = 0.70, *SD* = 0.43) scored significantly higher than those in the time pressure condition (*M* = 0.44, *SD* = 0.45), *p* = 0.033. In the 6-year-old group, the difference in helping behavior between the time delay condition (*M* = 0.81, *SD* = 0.32) and the time pressure condition (*M* = 0.58, *SD* = 0.43) was marginally significant, *p* = 0.066.

### Regression analysis

Since the ANOVA revealed significant main effects of both age group and time pressure, a simple effects analysis showed significant differences between the 4-year-old and 5-year-old groups under different time pressure conditions. Additionally, the Levene’s test indicated that the assumption of homogeneity of variance was met, *F*(5, 147) = 1.26, *p* = 0.285. Therefore, experiment 2 further employed a multiple linear regression model to analyze the effects of age (as a continuous variable) and time pressure on children’s helping behavior scores.

**Figure 3:**
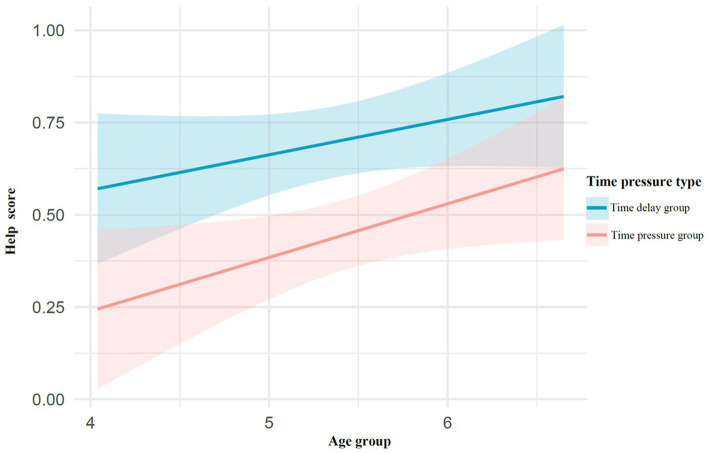
The effect of time pressure on children’s helping behavior.

The linear regression analysis revealed that the model was significant, *F*(2, 150) = 9.65, *p* < 0.001, explaining 11.4% of the total variance (adjusted *R^2^* = 0.10). A multiple linear regression was conducted to examine the effects of age and time pressure on the dependent variable. Specifically, age showed a significant positive predictive effect on children’s helping behavior scores [β = 0.12, *t* (150) = 2.45, *p* = 0.015, 95%CI = (0.02, 0.22)], In contrast, time pressure exhibited a significant negative predictive effect on helping behavior scores [β = −0.26, *t* (150) = −3.74, *p* < 0.001, 95%CI = (−0.39, −0.12)], as shown in Figure 3.

### Discussion

The results of experiment 1 indicated that children in the time pressure group exhibited significantly reduced helping behavior compared to those in the time delay group, supporting H1: time pressure reduces children’s helping behavior by inhibiting the reflective system. This finding provides new empirical evidence for the application of the dual-process theory in the domain of children’s helping behavior, suggesting that children’s helping behavior relies more on the cognitive processing of the reflective system rather than the automatic responses of the intuitive system (Evans and Stanovich, 2013).

From the perspective of the dual-process theory, time pressure inhibits the functioning of the reflective system by limiting the time available for deep processing (Evans and Curtis-Holmes, 2005). Helping behavior requires actively recognizing others’ needs (goal-directedness) and coordinating multi-step actions (execution complexity) (Paulus, 2014), which heavily depends on the cognitive processing of the reflective system. Time pressure disrupts the complete cognitive process from need recognition to behavioral execution. Combined with the theory of limited cognitive resources (Baumeister and Vohs, 2018), time pressure significantly increases children’s cognitive load, leading to the depletion of cognitive resources originally allocated for helping decisions. This mechanism aligns with existing research, which shows that time pressure impairs cognitive functioning and decision quality (Sussman and Sekuler, 2022). For young children, whose cognitive resources and self-control abilities are still in the developmental stage (Diamond, 2013), the cognitive load induced by time pressure is more likely to exceed their resource capacity. Notably, the results of experiment 1 contrast with findings in adult studies, where time pressure promotes cooperative behavior (Rand et al., 2012), but are consistent with findings in adolescent studies (Nava et al., 2023). This discrepancy reflects the cognitive processing characteristics at different developmental stages: adults have established stable decision-making patterns for helping behavior, whereas young children are in a critical period of transition from situational dependence to active regulation in helping behavior (Paulus et al., 2020).

The results of experiment 1 also revealed a moderating effect of age on the impact of time pressure: the reduction in helping behavior under time pressure was smaller for 6-year-olds compared to the 4- and 5-year-old groups. This finding aligns with the study by Plötner et al. (2021), which showed that the decrease in sharing behavior under time pressure for 7-year-olds was only one-third of that observed in 3- to 5-year-olds, suggesting that age-related cognitive maturation may buffer the negative effects of time pressure. As children grow older, the helping behavior of 6-year-olds becomes increasingly stable, selective, and goal-directed (Paulus et al., 2020). By this age, children begin to develop more advanced cognitive strategies, enabling them to better balance time pressure with helping decisions (Zelazo et al., 2021). This finding indicates that with age, children’s ability to regulate cognitive resources gradually improves, allowing them to better mitigate the negative impact of time pressure on helping behavior.

The results of experiment 1 indicate that time pressure significantly reduces children’s helping behavior by inhibiting the reflective system, with a more pronounced effect on 4- to 5-year-old children. While experiment 1 revealed the mechanism through which external time pressure influences children’s helping behavior, time pressure, as an external situational factor, may be closely related to the internal cognitive resource state of children. Previous research has shown that self-control resources, as a critical component of cognitive resources, play an important role in the occurrence of prosocial behavior (Baumeister and Vohs, 2018). Studies have suggested that the depletion of self-control resources may influence prosocial behavior through dual pathways: it can either weaken the regulatory function of the reflective system (Meng and Moriguchi, 2021) or enhance the automatic responses of the intuitive system (Ugur, 2021). Compared to time pressure, ego depletion, as an internal factor, may affect the occurrence of helping behavior through different mechanisms. Therefore, experiment 2 will systematically examine the mechanisms by which ego depletion, as an internal factor, influences children’s helping behavior.

## Experiment 2: the impact of ego depletion on children’s helping behavior in 4–6-year-old children

### Purpose

From the perspective of internal factors, experiment 2 explores the mechanism by which ego depletion affects helping behavior in 4- to 6-year-old children, using a ego depletion task combined with the pencil-picking helping paradigm.

### Methods

#### Participants

Using GPower 3.1 (Faul et al., 2009), it was calculated that 128 participants would be needed to ensure sufficient statistical power (1–β > 0.8) under the condition of a medium effect size (effect size *f* = 0.25). This study recruited a total of 221 participants aged 4 to 6 years from a region in central China. Specifically, our sample included 76 4-year-olds (*M* = 4.49 years, *SD* = 0.24 years), 74 5-year-olds (*M* = 5.50 years, *SD* = 0.28 years), and 71 6-year-olds (*M* = 6.30 years, *SD* = 0.17 years). All participants were randomly assigned to either the high-depletion group or the low-depletion group. Informed consent forms were signed by all participants’ parents or guardians, and the study was approved by the ethics committee of the affiliated institution.

#### Experimental design

**Figure 4:**
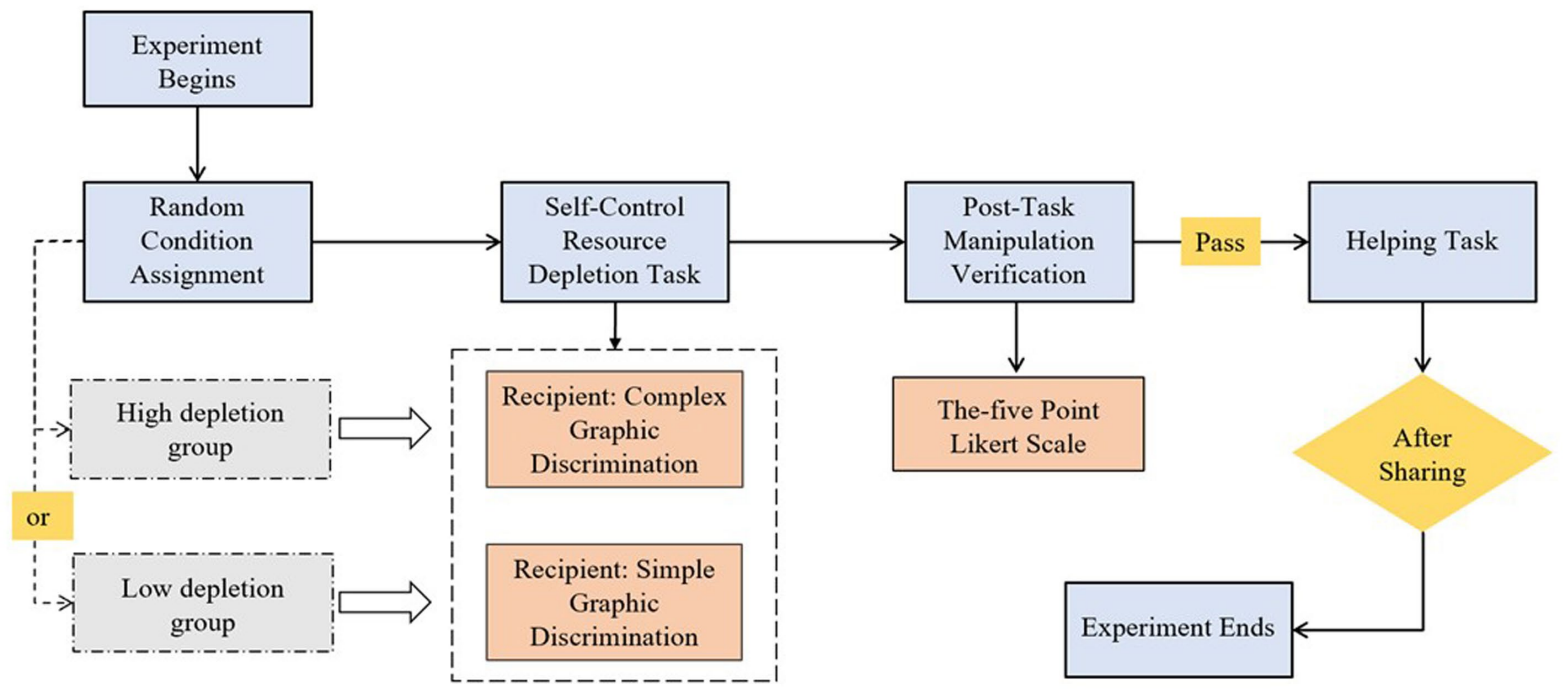
Flowchart of experiment 2.

Experiment 2 employed a 2 (ego depletion: high depletion, low depletion) × 3 (age: 4, 5, 6) between-subjects experimental design, where ego depletion and age served as independent variables, and the dependent variable was the score of helping behavior (Figure 4).

#### Procedure

This study used a modified stop-signal task to assign participants to ego depletion groups, consisting of the following steps:

1. Experiment Preparation. Same as in experiment 1.
2. Grouping. Children were randomly assigned to either the high-depletion group or the low-depletion group.
3. Ego depletion Task. The high-depletion group completed the stop-signal task, where children were required to press the space bar when a wolf appeared alone but to inhibit pressing the space bar when a rabbit appeared after the wolf. The low-depletion group completed a simple shape judgment task, where children only needed to press the space bar when the wolf appeared.
4. Post-Task Operability Test. Children rated their level of resource depletion using a 5-point Likert scale. The experimenter presented the children with graphs of 1 to 5 frames of electricity, corresponding to the range from very tired to very relaxed, and asked them, “Do you feel tired after doing the task you just did? Point out which graph you think better represents your current state.”
5. Helping Task. Same as in experiment 1.
6. End of Experiment. Same as in experiment 1.

#### Data processing and analysis

Data Coding: Same as in experiment 1.

Data Analysis: All data from experiment 2 will be processed and analyzed using R software (version 4.4.2). A two-way analysis of variance (Two-Way ANOVA) will be conducted in experiment 1 to examine the main effects and interaction effects of age (4 years, 5 years, 6 years) and ego depletion (high, low) on children’s helping behavior scores. If the ANOVA reveals significant main effects or interaction effects (*p* < 0.05), Tukey’s HSD will be used for *post hoc* multiple comparisons. Additionally, to further explore the predictive effects of age (as a continuous variable) and ego depletion on children’s helping behavior, multiple linear regression analysis will be performed.

### Results

#### Operability testing

Before conducting data analysis, tests for normality and homogeneity of variance were performed. The results of the normality test indicated that the fatigue scores of all groups significantly deviated from a normal distribution (Shapiro–Wilk test), high-depletion group: *W* = 0.80, low-depletion group: *W* = 0.73. The homogeneity of variance test revealed that the assumption of homogeneity of variances was not satisfied, *F*(5, 215) = 7.41, *p* = 0.007. Given that the data did not meet the basic assumptions of parametric tests, the Kruskal-Wallis rank-sum test, a non-parametric method, was used to analyze the effect of ego depletion on children’s fatigue scores. The Kruskal-Wallis rank-sum test results showed a significant difference in fatigue scores between the ego depletion groups, χ^2^(1) = 28.363, *p* < 0.001. *Post hoc* Dunn test results further indicated that the high-depletion group scored significantly higher than the low-depletion group (*p* < 0.001). These findings confirm the successful manipulation of ego depletion in this study.

#### Preliminary data analysis

In the low depletion group, helping behavior scores increased with age, with 6-year-olds scoring the highest (*M* = 0.77, *SD* = 0.39), followed by 5-year-olds (*M* = 0.74, *SD* = 0.41) and 4-year-olds (*M* = 0.70, *SD* = 0.43). A similar age-related trend was observed in the high depletion group, where 6-year-olds achieved the highest scores (*M* = 0.65, *SD* = 0.48), followed by 5-year-olds (*M* = 0.47, *SD* = 0.49) and 4-year-olds (*M* = 0.22, *SD* = 0.38), as shown in Table 2.

**Table 2:** Descriptive statistics on the outcome of helping behavior of young children under different conditions of resource depletion.

Age group	Depletion type	*n*	*M ± SD*
4 years old group	High depletion group	38	0.224 ± 0.380
	Low depletion group	38	0.697 ± 0.428
5 years old group	High depletion group	39	0.474 ± 0.486
	Low depletion group	35	0.743 ± 0.409
6 years old group	High depletion group	36	0.653 ± 0.475
	Low depletion group	35	0.771 ± 0.390

#### Analysis of variance

**Figure 5:**
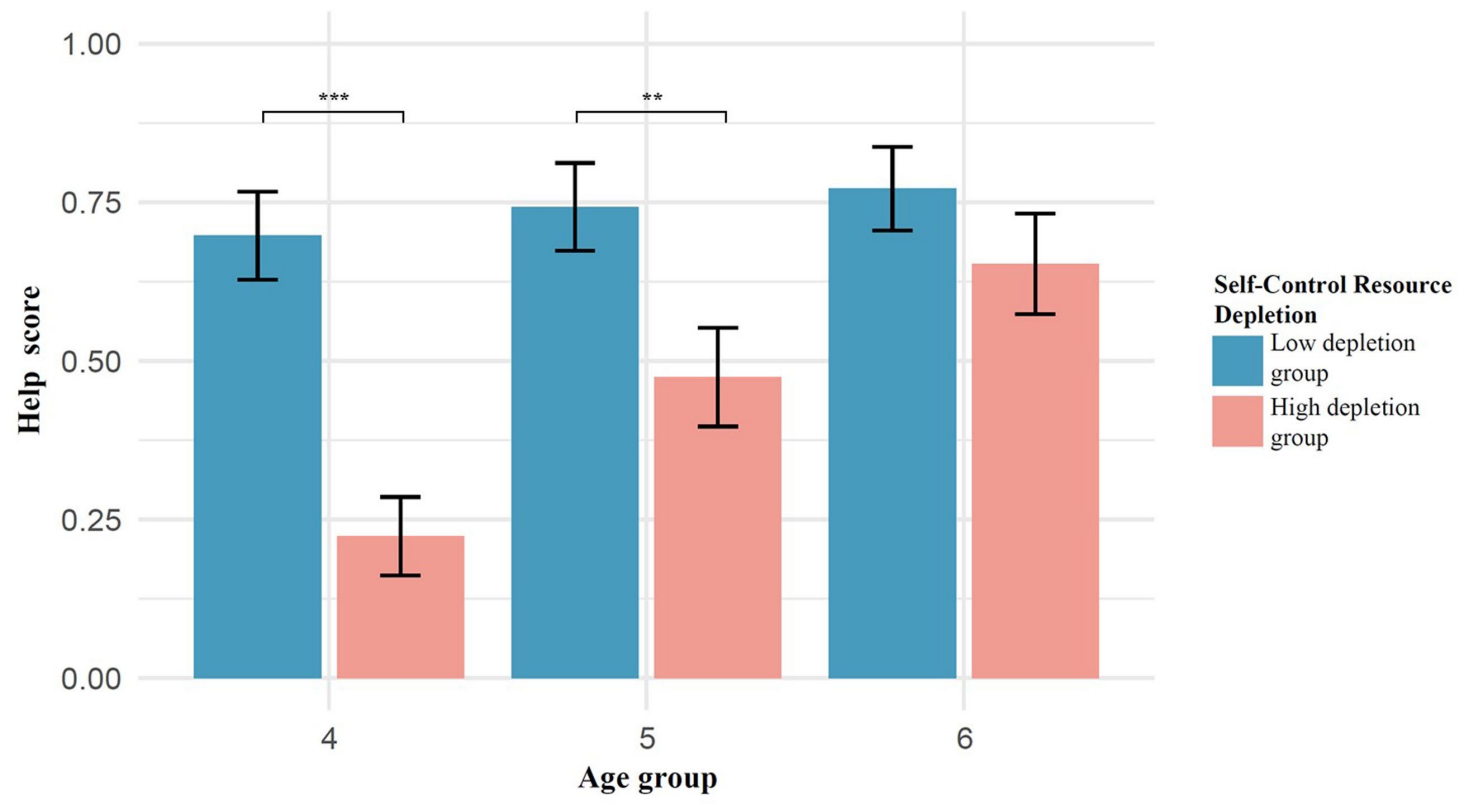
Differences in helping behavior of children under different ego depletion conditions.

Using helping behavior scores as the dependent variable, a 2 (ego depletion: high depletion group, low depletion group) × 3 (age: 4, 5, 6) two-way analysis of variance (ANOVA) was conducted. The results showed that age had a significant effect on helping behavior scores, with a significant main effect of age, *F* (2, 218) = 6.42, *p* = 0.002, η^2^_p_ = 0.056. Post hoc multiple comparisons revealed that the helping behavior of 4-year-olds was significantly lower than that of 6-year-olds (*p* = 0.0014), while the differences between 5-year-olds and 4-year-olds (*p* = 0.091) and between 5-year-olds and 6-year-olds (*p* = 0.319) were not significant. The main effect of ego depletion was also significant, *F* (1, 219) = 24.91, *p* < 0.001, η^2^_p_ = 0.104. Under the high depletion group (*M* = 0.45, *SD* = 0.48), children’s helping behavior was significantly lower than under the low depletion group (*M* = 0.74, *SD* = 0.41). The difference between the low depletion group and the high depletion group was highly significant (*p* < 0.001). The interaction effect between ego depletion and age was significant, *F* (5, 215) = 3.16, *p* = 0.045, η^2^_p_ = 0.029, as shown in Figure 5.

Simple effects analysis revealed that under the low depletion condition, there were no significant differences in helping behavior among 4-year-olds, 5-year-olds, and 6-year-olds (*ps* > 0.05). However, under the high depletion condition, the helping behavior of 4-year-olds was significantly lower than that of 5-year-olds (*p* = 0.0304) and 6-year-olds (*p* < 0.001), while the difference between 5-year-olds and 6-year-olds was not significant (*p* = 0.17). In the 4-year-old group, children in the low depletion group (*M* = 0.70, *SD* = 0.43) exhibited significantly higher helping behavior than those in the high depletion group (*M* = 0.22, *SD* = 0.38), *p* < 0.001; in the 5-year-old group, children in the low depletion group (*M* = 0.74, *SD* = 0.41) also demonstrated significantly higher helping behavior than those in the high depletion group (*M* = 0.47, *SD* = 0.49), *p* = 0.008; in contrast, in the 6-year-old group, there was no significant difference in helping behavior between the low depletion group (*M* = 0.77, *SD* = 0.39) and the high depletion group (*M* = 0.65, *SD* = 0.48), *p* = 0.247.

### Regression analysis

Since the results of the ANOVA indicated significant main effects and interaction effects of age group and ego depletion, and the Levene’s test showed that the assumption of homogeneity of variance was met, *F* (5, 215) = 2.12, *p* = 0.065, experiment 2 further employed a multiple linear regression model to analyze the effects of age (as a continuous variable) and ego depletion on children’s helping behavior scores.

**Figure 6:**
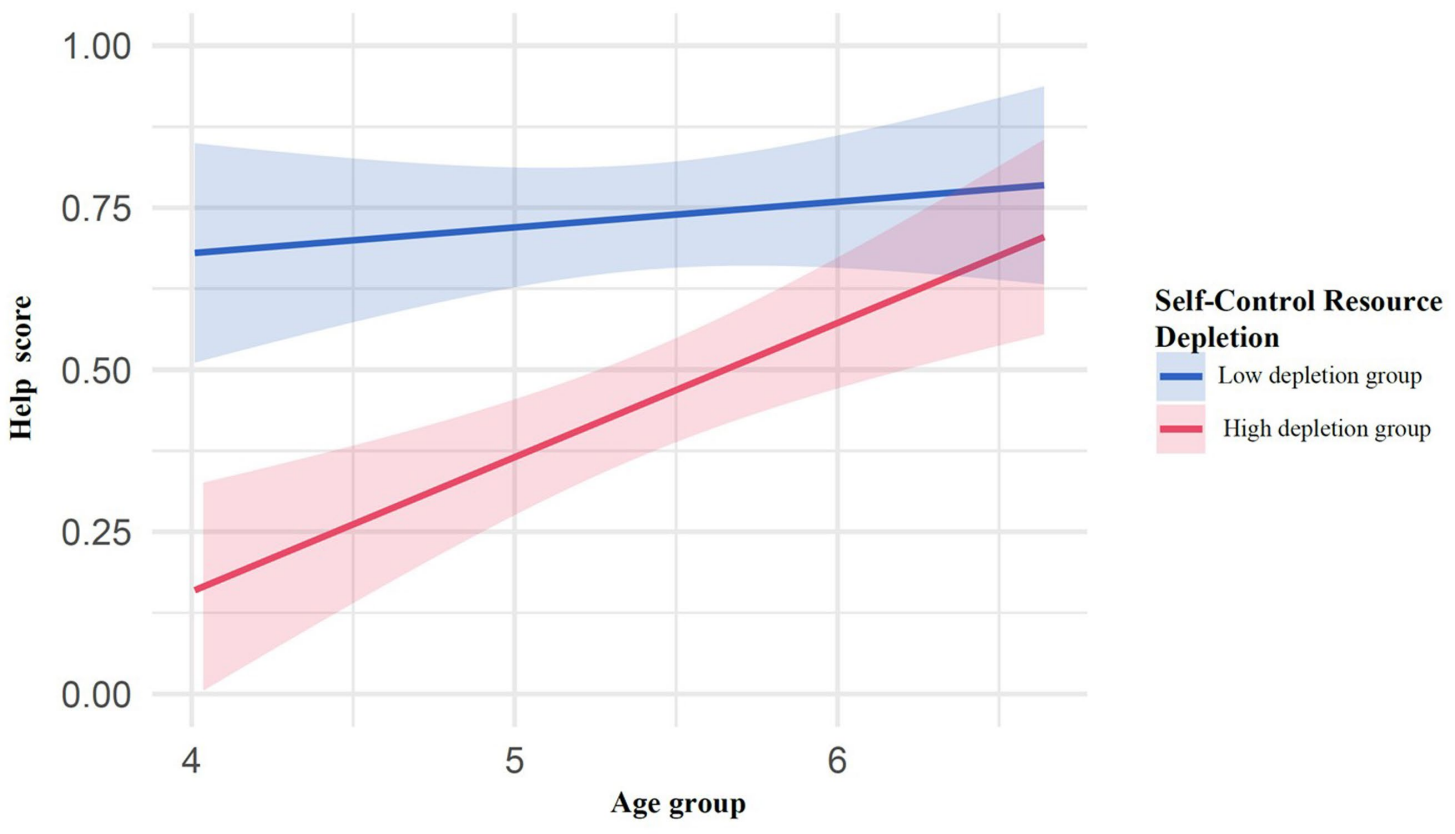
The effect of ego depletion on children’s helping behavior.

The linear regression analysis revealed that the overall model was significant, *F*(2, 218) = 17.71, *p* < 0.001, explaining 13.98% of the variance (adjusted *R^2^* = 0.132). Specifically, age had a significant positive predictive effect on children’s helping behavior scores [β = 0.13, *t* (218) = 3.33, *p* = 0.001, 95%CI = (0.05, 0.20)], while ego depletion had a significant negative predictive effect on helping behavior scores [β = −0.29, *t* (218) = −4.89, *p* < 0.001, 95%CI = (−0.40, −0.17)], as shown in Figure 6.

### Discussion

The results of experiment 2 indicated that compared to the low depletion group, children in the high ego depletion group exhibited significantly reduced helping behavior, supporting the research H2: ego depletion reduces children’s helping behavior by inhibiting the reflective system. This finding aligns with the theory of limited cognitive resources (Baumeister and Vohs, 2018) and supports the view that resource depletion weakens prosocial behavior. Resource depletion may reduce children’s cognitive resource reserves, suppressing the operation of the reflective system, thereby hindering the occurrence of helping behavior. This mechanism is similar to the impact of time pressure on children’s helping behavior observed in experiment 1, further demonstrating that the occurrence of helping behavior heavily relies on sufficient cognitive resource support.

The inhibitory effect of ego depletion on children’s helping behavior may stem from two aspects. First, it reflects the decision-making characteristics of children in situations with limited cognitive resources. Research has shown that ego depletion significantly increases children’s cognitive load, limiting their ability to process and interpret social cues (Diamond, 2013). Eisenberg et al.’s (2010) study indicated that ego depletion affects children’s emotion regulation, making it difficult for them to accurately recognize and respond to the needs of others. Second, this is closely related to the development of early social cognitive abilities in children. Between the ages of 4 and 6, children’s executive functions are undergoing rapid development, and ego depletion is more likely to lead them to rely on the intuitive system for decision-making rather than engaging in reflective thinking (Zelazo and Carlson, 2012).

The findings also revealed the moderating role of age in the impact of ego depletion. Under low depletion conditions, there were no significant differences in helping behavior across the three age groups, indicating that when cognitive resources are sufficient, even younger children can exhibit good helping behavior. However, under high depletion conditions, the helping behavior of 4-year-olds was significantly lower than that of 5-year-olds and 6-year-olds, while no significant difference was observed between the 5-year-olds and 6-year-olds. This pattern highlights the importance of cognitive development in coping with the effects of resource depletion: as children grow older, they gradually develop more effective cognitive strategies to mitigate the negative impact of depletion. With age, children’s ability to manage cognitive resources strengthens progressively. Meng and Moriguchi (2021) found that children’s sharing behavior significantly decreased after completing inhibitory control tasks, which aligns closely with the results of this study. Blair et al. (2014) pointed out that children’s self-regulation abilities develop progressively with age, explaining why older children can maintain relatively stable helping behavior under resource depletion conditions. Notably, for 6-year-olds, there was no significant difference in helping behavior between high and low depletion conditions, indicating that they had already developed strong cognitive resource regulation abilities (Zelazo et al., 2021). The inhibitory effect of ego depletion on children’s helping behavior exhibits developmental sensitivity—the younger the child, the stronger the negative impact of resource depletion.

The results of experiment 2 showed that ego depletion significantly inhibited children’s helping behavior, with a more pronounced effect on 4- to 5-year-old children. This finding further supports the critical role of the reflective system in prosocial behavior (Evans and Stanovich, 2013) and highlights the importance of age-related resource regulation abilities in mitigating the negative impact of resource depletion.

## General discussion

This study, based on the theory of limited cognitive resources (Baumeister, 2014) and the dual-process theory (Evans and Stanovich, 2013), systematically examined the mechanisms by which external time pressure and internal ego depletion affect the helping behavior of 4- to 6-year-old children through experiments 1 and 2.

### Both external time pressure and internal ego depletion inhibit children’s helping behavior

The results of both experiments consistently demonstrate that cognitive resource depletion (whether stemming from external time pressure or internal ego depletion) significantly reduces young children’s helping behavior. This inhibitory effect is primarily achieved through the following mechanisms: First, resource depletion directly limits the cognitive processing capacity of the deliberative system, making it difficult for children to adequately assess others’ needs and plan appropriate helping behaviors (Diamond, 2013). Second, resource depletion weakens the executive function’s ability to suppress selfish impulses (Baumeister and Vohs, 2018). This finding supports the core hypothesis of this study: children’s helping behavior relies more on the cognitive processing of the deliberative system rather than the automated responses of the intuitive system.

Time pressure may influence children’s helping decisions through two pathways: First, time constraints directly compress the cognitive processing window of the deliberative system, preventing children from fully evaluating others’ needs (Diamond, 2013). Second, the cognitive resource competition induced by time pressure undermines the executive function’s ability to suppress selfish impulses (Baumeister and Vohs, 2018). This mechanism is corroborated by Corbit et al.’s (2023) study on children’s cooperative behavior, which found that when cognitive resources are limited, children are more likely to prioritize immediate rewards over long-term social benefits. Under time pressure conditions, the reduction in children’s helping behavior indicates that they struggle to sufficiently mobilize cognitive resources to recognize others’ needs and take appropriate helping actions. This result further validates the theory of limited cognitive resources (Baumeister and Vohs, 2018), which posits that individuals’ cognitive resources are finite, and when these resources are occupied or depleted, decision-making quality significantly declines.

The inhibitory effect of ego depletion on young children’s helping behavior may primarily stem from two reasons: First, ego depletion restricts children’s behavioral flexibility, leading them to fail in promptly adjusting their behavioral strategies when faced with situations requiring help (Diamond, 2013; Zelazo and Müller, 2002). Second, when cognitive resources are depleted, children’s executive function is significantly impaired, manifesting as a decline in inhibitory control, making it difficult for them to effectively translate their intrinsic helping intentions into concrete prosocial behaviors (Carlson and Moses, 2001). This decline in executive function is further reflected in multiple aspects of helping behavior, including delays in the initiation of helping actions, reduced persistence in helping behavior, and diminished efficiency of the behavior (Kochanska et al., 2000). These findings suggest that ego depletion generally exerts a suppressive effect on young children’s helping behavior, with this effect being particularly pronounced in 4- and 5-year-old children.

### The moderating effect of age: time pressure and ego depletion primarily inhibit the helping behavior of 4- to 5-year-old children, but not 6-year-old children

Both experiments revealed a moderating effect of age: Cognitive resource depletion (whether caused by time pressure or ego depletion) had a greater impact on the helping behavior of 4- to 5-year-old children, while 6-year-old children demonstrated stronger resistance to interference. In experiment 1, the helping behavior of 6-year-old children under time pressure was significantly less affected compared to the 4- and 5-year-old groups. In experiment 2, ego depletion significantly inhibited the helping behavior of the 4- and 5-year-old groups, whereas the difference between high and low depletion conditions in the 6-year-old group was not significant.

Compared to 4- and 5-year-old children, 6-year-old children are less affected by time pressure and ego depletion, which may be attributed to significant advancements in their cognitive and emotional regulation abilities. On the one hand, as children grow older, their cognitive development progresses, with notable improvements in executive function and information processing capabilities (Blair et al., 2014; Zelazo et al., 2018). The cognitive development of 4- and 5-year-old children is still immature, leading to insufficient processing speed and depth, making them more susceptible to the effects of time pressure and cognitive resource depletion. In contrast, 6-year-old children may have developed more advanced executive functions and cognitive resource allocation abilities, enabling them to better sustain attention and integrate multiple social cues, thereby maintaining helping behavior even under resource-limited conditions (Pushparatnam et al., 2021; Diamond, 2013). On the other hand, as children age, their emotional recognition and regulation abilities gradually improve (Denham et al., 2020). The ages of 4 to 6 represent a critical period for the development of prosocial behavior, with age 6 marking a qualitative leap in cognitive regulation abilities (Zelazo and Carlson, 2012). The moderating effect of age on the influence of time pressure and ego depletion reflects the dynamic process of cognitive system development in young children (Best and Miller, 2010). These findings indicate that 6-year-old children are capable of exhibiting stable helping behavior even under external time pressure and internal cognitive resource depletion. This suggests that young children’s helping behavior is not merely a simple intuitive response but requires the deep cognitive processing of the deliberative system.

## Limitations and suggestions for future research

This study explored the effects of time pressure (an external factor) and ego depletion (an internal factor) on the helping behavior of 4- to 6-year-old children through two experiments. The findings revealed that both factors significantly inhibited children’s helping behavior, with 4- to 5-year-old children being more affected, while 6-year-old children demonstrated stronger resistance to interference. These results suggest that helping behavior relies on the deep cognitive processing of the deliberative system. However, this study has several limitations: First, helping behavior was measured solely using the pen-picking task, which may underestimate children’s prosocial behavior in diverse contexts (Paulus, 2014). Second, the sample was limited to 4- to 6-year-old children from a specific region, restricting the cultural generalizability of the findings. Furthermore, the study did not examine potential mediating variables, such as emotional states or empathy (Diamond, 2013), and its cross-sectional design prevents the investigation of the long-term dynamics of behavioral development.

Future research could employ more diverse measurement methods (e.g., combining naturalistic observation with physiological indicators) and expand the age and cultural range of the sample to explore the moderating effect of East Asian collectivist values on helping behavior. Additionally, future studies should delve deeper into mechanisms such as emotional regulation and empathy and use longitudinal designs to uncover the long-term effects of cognitive resource depletion on helping behavior as well as individual differences.

## Data Availability

The original contributions presented in the study are included in the article/Supplementary material, further inquiries can be directed to the corresponding author.

## References

[ref1] Aknin L. B. Hamlin J. K. Dunn E. W. (2012). Giving leads to happiness in young children. PLoS One 7:e39211. doi: 10.1371/journal.pone.0039211, PMID: 22720078 PMC3375233

[ref7001] Baumeister R. F. (2014). Self-regulation, ego depletion, and inhibition. Neuropsychologia 65, 313–319. 25149821 10.1016/j.neuropsychologia.2014.08.012

[ref2] Baumeister R. F. (2002). Ego depletion and self-control failure: an energy model of the self's executive function. Self Identity 1, 129–136. doi: 10.1080/152988602317319302

[ref3] Baumeister R. F. Vohs K. D. (2018). “Strength model of self-regulation as limited resource: assessment, controversies, update” in Self-regulation and self-control. ed. Baumeister R. F. (Abingdon: Routledge), 78–128.

[ref4] Best J. R. Miller P. H. (2010). A developmental perspective on executive function. Child Dev. 81, 1641–1660. doi: 10.1111/j.1467-8624.2010.01499.x, PMID: 21077853 PMC3058827

[ref5] Blair C. Raver C. C. Berry D. J. (2014). Two approaches to estimating the effect of parenting on the development of executive function in early childhood. Dev. Psychol. 50, 554–565. doi: 10.1037/a0033647, PMID: 23834294 PMC4682354

[ref6] Brownell C. A. Svetlova M. Anderson R. Nichols S. R. Drummond J. (2013). Socialization of early prosocial behavior: parents’ talk about emotions is associated with sharing and helping in toddlers. Infancy 18, 91–119. doi: 10.1111/j.1532-7078.2012.00125.x 23264753 PMC3524590

[ref7] Carlson S. M. Koenig M. A. Harms M. B. (2013). Theory of mind. Wiley Interdiscip. Rev. Cogn. Sci. 4, 391–402. doi: 10.1002/wcs.1232, PMID: 26304226

[ref8] Carlson S. M. Moses L. J. (2001). Individual differences in inhibitory control and children's theory of mind. Child Dev. 72, 1032–1053. doi: 10.1111/1467-8624.00333, PMID: 11480933

[ref9] Corbit J. Dockrill M. Hartlin S. Moore C. (2023). Intuitive cooperators: time pressure increases children's cooperative decisions in a modified public goods game. Dev. Sci. 26:e13344. doi: 10.1111/desc.13344, PMID: 36399363

[ref10] Denham S. A. Ferrier D. E. Howarth G. Z. Herndon K. J. Bassett H. H. (2020). “Key considerations in assessing young children’s emotional competence” in Social and emotional learning. ed. Denham S. A. (Abingdon: Routledge), 27–45.

[ref11] DeWall C. N. Baumeister R. F. Gailliot M. T. Maner J. K. (2008). Depletion makes the heart grow less helpful: helping as a function of self-regulatory energy and genetic relatedness. Personal. Soc. Psychol. Bull. 34, 1653–1662. doi: 10.1177/0146167208323981, PMID: 19050337

[ref12] Diamond A. (2013). Executive functions. Annu. Rev. Psychol. 64, 135–168. doi: 10.1146/annurev-psych-113011-143750 23020641 PMC4084861

[ref13] Dunfield K. A. (2014). A construct divided: prosocial behavior as helping, sharing, and comforting subtypes. Front. Psychol. 5:958. doi: 10.3389/fpsyg.2014.00958 25228893 PMC4151454

[ref14] Eisenberg N. Cumberland A. Spinrad T. L. (1998). Parental socialization of emotion. Psychol. Inq. 9, 241–273. doi: 10.1207/s15327965pli0904_1, PMID: 16865170 PMC1513625

[ref15] Eisenberg N. Spinrad T. L. Eggum N. D. (2010). Emotion-related self-regulation and its relation to children's maladjustment. Annu. Rev. Clin. Psychol. 6, 495–525. doi: 10.1146/annurev.clinpsy.121208.131208 20192797 PMC3018741

[ref16] Eisenberg N. Spinrad T. L. Morris A. S. (2013). “13 Prosocial Development” in The Oxford Handbook of Developmental Psychology, Vol. 1: Body and Mind. ed. Eisenberg N. (Oxford, UK: Oxford University Press).

[ref17] Eisenberg N. Spinrad T. Sadovsky A. (2006). “Empathy-related responding in children” in Handbook of moral development. eds. Killen M. Smetana J. G. (London: Psychology Press), 535–568.

[ref18] Evans J. S. B. Curtis-Holmes J. (2005). Rapid responding increases belief bias: evidence for the dual-process theory of reasoning. Think. Reason. 11, 382–389. doi: 10.1080/13546780542000005, PMID: 40101104

[ref19] Evans J. S. B. Stanovich K. E. (2013). Dual-process theories of higher cognition: advancing the debate. Perspect. Psychol. Sci. 8, 223–241. doi: 10.1177/1745691612460685, PMID: 26172965

[ref20] Everett J. A. Ingbretsen Z. Cushman F. Cikara M. (2017). Deliberation erodes cooperative behavior—even towards competitive out-groups, even when using a control condition, and even when eliminating selection bias. J. Exp. Soc. Psychol. 73, 76–81. doi: 10.1016/j.jesp.2017.06.014

[ref22] Faul F. Erdfelder E. Buchner A. Lang A. G. (2009). Statistical power analyses using G* power 3.1: tests for correlation and regression analyses. Behav. Res. Methods 41, 1149–1160. doi: 10.3758/BRM.41.4.1149, PMID: 19897823

[ref23] Gärtner M. (2018). The prosociality of intuitive decisions depends on the status quo. J. Behav. Exp. Econ. 74, 127–138. doi: 10.1016/j.socec.2018.04.005, PMID: 40147767

[ref24] Halali E. Bereby-Meyer Y. Ockenfels A. (2013). Is it all about the self? The effect of self-control depletion on ultimatum game proposers. Front. Hum. Neurosci. 7:240. doi: 10.3389/fnhum.2013.00240, PMID: 23781182 PMC3680729

[ref25] Hay D. F. Paine A. L. Perra O. Cook K. V. Hashmi S. Robinson C.. (2021). Prosocial and aggressive behavior: a longitudinal study. Monogr. Soc. Res. Child Dev. 86, 7–103. doi: 10.1111/mono.12427, PMID: 33973244 PMC9943493

[ref26] Jarke-Neuert J. Lohse J. (2022). I’m in a hurry, I don't want to know! Strategic ignorance under time pressure. J. Exp. Psychol. Gen. 151, 2833–2845. doi: 10.1037/xge0001222, PMID: 35737530

[ref27] Katerkamp A. Horn L. (2025). Preschoolers' prosocial behavior in groups—testing effects of dominance, popularity, and friendship. Front. Psychol. 16:1478493. doi: 10.3389/fpsyg.2025.1478493 40008342 PMC11850581

[ref28] Kochanska G. Murray K. T. Harlan E. T. (2000). Effortful control in early childhood: continuity and change, antecedents, and implications for social development. Dev. Psychol. 36, 220–232. doi: 10.1037/0012-1649.36.2.220, PMID: 10749079

[ref29] Lohse J. Goeschl T. Diederich J. H. (2017). Giving is a question of time: response times and contributions to an environmental public good. Environ. Resour. Econ. 67, 455–477. doi: 10.1007/s10640-016-0029-z

[ref30] Martin A. Olson K. R. (2013). When kids know better: paternalistic helping in 3-year-old children. Dev. Psychol. 49, 2071–2081. doi: 10.1037/a0031715, PMID: 23379296

[ref31] Martinsson P. Myrseth K. O. R. Wollbrant C. (2012). Reconciling pro-social vs. selfish behavior: on the role of self-control. Judgm. Decis. Mak. 7, 304–315. doi: 10.1017/S1930297500002278

[ref32] Meng X. Moriguchi Y. (2021). Neural basis for egalitarian sharing in five-to six-year-old children. Neuropsychologia 154:107787. doi: 10.1016/j.neuropsychologia.2021.107787, PMID: 33577876

[ref33] Nava F. Margoni F. Herath N. Nava E. (2023). Age-dependent changes in intuitive and deliberative cooperation. Sci. Rep. 13:4457. doi: 10.1038/s41598-023-31691-9, PMID: 36932178 PMC10023788

[ref34] Paulus M. (2014). The early origins of human charity: developmental changes in preschoolers’ sharing with poor and wealthy individuals. Front. Psychol. 5:344. doi: 10.3389/fpsyg.2014.00344 25018735 PMC4071819

[ref35] Paulus M. Christner N. Wörle M. (2020). The normative status of friendship: do young children enforce sharing with friends and appreciate reasonable partiality? J. Exp. Child Psychol. 194:104826. doi: 10.1016/j.jecp.2020.104826, PMID: 32179294

[ref36] Plötner M. Hepach R. Over H. Carpenter M. Tomasello M. (2021). Young children share more under time pressure than after a delay. PLoS One 16:e0248121. doi: 10.1371/journal.pone.0248121, PMID: 33724998 PMC7963052

[ref37] Pushparatnam A. Luna Bazaldua D. A. Holla A. Azevedo J. P. Clarke M. Devercelli A. (2021). Measuring early childhood development among 4–6 year olds: the identification of psychometrically robust items across diverse contexts. Front. Public Health 9:569448. doi: 10.3389/fpubh.2021.569448 33614575 PMC7888256

[ref38] Rand D. G. Greene J. D. Nowak M. A. (2012). Spontaneous giving and calculated greed. Nature 489, 427–430. doi: 10.1038/nature11467, PMID: 22996558

[ref39] Schulz J. F. Fischbacher U. Thöni C. Utikal V. (2014). Affect and fairness: dictator games under cognitive load. J. Econ. Psychol. 41, 77–87. doi: 10.1016/j.joep.2012.08.007, PMID: 40147767

[ref40] Shah A. K. Mullainathan S. Shafir E. (2012). Some consequences of having too little. Science 338, 682–685. doi: 10.1126/science.1222426, PMID: 23118192

[ref41] Sierksma J. Shutts K. (2021). Competence-based helping: Children’s consideration of need when providing others with help. J. Exp. Child Psychol. 210:105206. doi: 10.1016/j.jecp.2021.105206 34134018 PMC13274732

[ref42] Sinha I. Smith M. F. (2000). Consumers' perceptions of promotional framing of price. Psychol. Mark. 17, 257–275. doi: 10.1002/(SICI)1520-6793(200003)17:3<257::AID-MAR4>3.0.CO;2-P

[ref43] Steinbeis N. (2018). Taxing behavioral control diminishes sharing and costly punishment in childhood. Dev. Sci. 21:e12492. doi: 10.1111/desc.12492, PMID: 28032451

[ref44] Sussman R. F. Sekuler R. (2022). Feeling rushed? Perceived time pressure impacts executive function and stress. Acta Psychol. 229:103702. doi: 10.1016/j.actpsy.2022.103702, PMID: 35985154 PMC9506568

[ref45] Ugur Z. B. (2021). Does self-control foster generosity? Evidence from ego depleted children. J. Behav. Exp. Econ. 90:101652. doi: 10.1016/j.socec.2020.101652

[ref46] Warneken F. Tomasello M. (2006). Altruistic helping in human infants and young chimpanzees. Science 311, 1301–1303. doi: 10.1126/science.1121448, PMID: 16513986

[ref47] Wellman H. M. Liu D. (2004). Scaling of theory-of-mind tasks. Child Dev. 75, 523–541. doi: 10.1111/j.1467-8624.2004.00691.x 15056204

[ref48] Zelazo P. D. Carlson S. M. (2012). Hot and cool executive function in childhood and adolescence: development and plasticity. Child Dev. Perspect. 6, 354–360. doi: 10.1111/j.1750-8606.2012.00246.x

[ref7002] Zelazo P. D. Forston J. L. Masten A. S. Carlson S. M. (2018). Mindfulness plus reflection training: Effects on executive function in early childhood. Frontiers in psychology. 9:208. 29535661 10.3389/fpsyg.2018.00208 PMC5834482

[ref49] Zelazo P. D. Lourenco S. F. Frank M. C. Elison J. T. Heaton R. K. Wellman H. M.. (2021). Measurement of cognition for the National Children's study. Front. Pediatr. 9:603126. doi: 10.3389/fped.2021.603126, PMID: 34136435 PMC8200393

[ref50] Zelazo P. D. Müller U. (2002). “Executive function in typical and atypical development” in Blackwell handbook of childhood cognitive development. ed. Zelazo P. D. (Hoboken, NJ: Wiley), 445–469.

